# *Lactobacillus plantarum* surface-displayed FomA (*Fusobacterium nucleatum*) protein generally stimulates protective immune responses in mice

**DOI:** 10.3389/fmicb.2023.1228857

**Published:** 2023-09-20

**Authors:** Xiaoyu Zhang, Huijie Xiao, Huaiyu Zhang, Yang Jiang

**Affiliations:** ^1^Department of Gastrointestinal and Colorectal Surgery, China-Japan Union Hospital of Jilin University, Changchun, China; ^2^Department of Rehabilitation Medicine, China-Japan Union Hospital of Jilin University, Changchun, China

**Keywords:** *F. nucleatum*, FomA, recombinant, *L. plantarum*, IBD

## Abstract

A significant correlation is observed between *Fusobacterium nucleatum* (*F. nucleatum*) and the evolution of inflammatory bowel disease (IBD). Particularly, FomA, a critical pathogenic element of *F. nucleatum*, inflicts substantial detriment to human intestinal health. Our research focused on the development of recombinant *Lactobacillus plantarum* that expresses FomA protein, demonstrating its potential in protecting mice from severe IBD induced by *F*. *nucleatum*. To commence, two recombinant strains, namely *L. plantarum* NC8-pSIP409-pgsA'-FomA and NC8-pSIP409-FnBPA-pgsA'-FomA, were successfully developed. Validation of the results was achieved through flow cytometry, ELISA, and MTT assays. It was observed that recombinant *L. plantarum* instigated mouse-specific humoral immunity and elicited mucosal and T cell-mediated immune responses. Significantly, it amplified the immune reaction of B cells and CD4^+^T cells, facilitated the secretion of cytokines such as IgA, IL4, and IL10, and induced lymphocyte proliferation in response to FomA protein stimulation. Finally, we discovered that administering recombinant *L. plantarum* could protect mice from severe IBD triggered by *F. nucleatum*, subsequently reducing pathological alterations and inflammatory responses. These empirical findings further the study of an innovative oral recombinant *Lactobacillus* vaccine.

## Introduction

*Fusobacterium nucleatum* (*F. nucleatum*), a Gram-negative, non-spore-forming anaerobic bacillus, is categorized into four subspecies: *nucleatum, polymorphum, vincentii (fusiforme)*, and *animalis* (Han, 2015; Nie et al., 2015). This bacterium inhabits not only the human oral cavity but also the digestive tract. Inflammatory bowel disease (IBD) is a disorder, which involves the gastrointestinal (GI) tract consisting of Crohn's disease (CD) and ulcerative colitis (UC; Khoramjoo et al., 2022). Recent studies have discovered a substantial correlation between *F. nucleatum* and the emergence of IBD (Allen-Vercoe et al., 2011; Liu et al., 2020). FomA, the major outer membrane pore protein of *F. nucleatum*, assists the bacterium in evading host immune surveillance by binding to human immunoglobulin's Fc fragments, thus establishing itself as a crucial pathogenic factor (Ponath et al., 2021). Furthermore, FomA acts as an efficient antigen, stimulating specific immune responses and potentially serving as an effective target to curb other pathogenic bacteria (Martin-Gallausiaux et al., 2020). Evidently, proteins such as FomA, expressed in the outer membrane of *E. coli*, can stimulate the body to produce specific antibodies (Claesson et al., 1990). Therefore, FomA is not only an effective immunogen but also a potential target for inhibiting *F. nucleatum*.

*Lactic acid bacteria* (*LAB*), a non-pathogenic probiotic species, exhibits significant benefits in preserving intestinal homeostasis and augmenting immunity (Reuben et al., 2019; De Filippis et al., 2020). *Lactobacillus plantarum* (*L. plantarum*), an acid and bile salt-resistant probiotic, adheres to intestinal epithelial cells, colonizing the gut (Kato et al., 1999). *L. plantarum* NC8, isolated from silage, shows a high propensity to express exogenous proteins, marking it as a promising candidate for an oral vaccine vector (Yang et al., 2017a,b; Kazemifard et al., 2022). FnBPA is located on the surface of *Staphylococcus aureus* (*SA*), where it interacts with the integrin α5β1 on the cell surface to adhere to the cell firmly. When *Lactococcus lactis* expresses FnBPA on its surface, it can bind to host cell surface receptors, allowing the recombinant bacteria to infect cells and achieve the delivery of exogenous DNA (Liu et al., 2018). In the early stages of *SA* infection, cell surface integrin α5β1 interacts with fibronectin-binding proteins (FnBPA and FnBPB) on the bacterial surface, firmly binding to endothelial cells (Sinha and Herrmann, 2005). This interaction depends on the bridge between FnBPs and integrins—the extracellular matrix protein Fn (Massey et al., 2001; Edwards et al., 2010). Soluble Fn is a dimeric sugar protein that contains different structural domains, each containing multiple templates, composed of fewer than 100 amino acid modules, and divided into type I, II, and III repeat sequences (Prystopiuk et al., 2018). FnBPA contains 11 repeat sequences that can bind to Fn, which, under the action of a tandem β-chain, can specifically bind to the four sequence modules in the N-terminal FI region (Schwarz-Linek et al., 2003). Six of these sequences have a high affinity (Edwards et al., 2010; Dufrêne, 2015), and any one of them can infect endothelial cells once bound to integrin α5β1 (Jin et al., 2018). The main role of integrin α5β1 in host cells is to enhance the adhesion of *SA* to host cells (Dufrêne, 2015; Xiao and Dufrêne, 2016). As such, when FnBPA is located on the surface of *L. plantarum*, it substantially enhances the invasion efficiency of somatic cells and ushers exogenous DNA fragments into host cells (Innocentin et al., 2009).

We utilized the truncated anchor sequence of poly-γ-glutamate synthase A' (PGSA'), a transmembrane protein from *Bacillus subtilis* known for its high display efficiency (Chen et al., 2021). Previous studies suggest that recombinant *L. plantarum* can serve as an oral vaccine, providing protective capabilities. Examples include *L. plantarum* used to express conserved M2E and HA2 fusion antigens to induce protection against influenza viruses (Yang et al., 2018b), the expression of the S-DCpep fusion protein against transmissible gastroenteritis virus (Yang et al., 2018a), and the improvement of the body's immune response through the expression of the porcine epidemic diarrhea virus S gene (Jin et al., 2018). Therefore, the development of a novel oral probiotic vaccine, using invasive *L. plantarum* expressing FnBPA and PGSA- as a vector, targeting FomA could offer a new breakthrough in treating severe IBD caused by *F. nucleatum*.

In this study, we engineered two recombinant *L. plantarum* vaccines expressing the FomA protein and assessed their capability to activate immune efficacy in mice. We investigate whether these vaccines demonstrated protective effects on mice suffering from severe IBD caused by *F. nucleatum*. For instance, the role of B-cell and T-cell activation is pivotal in the process of immune activation (Huszthy et al., 2019; Luo and Yin, 2021). Activated Th2 cells produce cytokines such as IL4, IL5, IL6, and IL10, while Th1 cells secrete IL2 and IFNγ, all of which promote the proliferation and differentiation of B cells into antibody-forming cells with synthesis and secretion functions (Romagnani, 2000). Some B cells even differentiate into memory cells. Our experiment also detected changes in the expression of Th1/2 type cytokines in mice, the levels of anti-specific antibodies in mice, and cellular and mucosal immune responses and lay a theoretical foundation for the development of oral vaccines.

## Materials and methods

### Bacterial strains, animals, and ethical statement

*Fusobacterium nucleatum* (337404, BNCC, China) was incubated in a brain heart immersion broth medium for 24 h at 37°C under anaerobic conditions. DSS (MP Biomedicals, Shanghai) was diluted 40 times. Invasive and non-invasive *L. plantarum* NC8 were kindly provided by Jing Liu (Jilin Agricultural University, Changchun, China) and grown in De Man, Rogosa, and Sharpe (MRS) media supplemented with erythromycin (10 μg/ml) at 30°C under anaerobic conditions. Compared with non-invasive *L. plantarum* NC8, the invasive *L. plantarum* strain expressing the FnBPA protein demonstrated increased adhesion and invasion rates in the mouse intestinal epithelial cell line IPEC-J2 (Liu et al., 2019).

Animals used in this experiment were purchased from HFK Bioscience Co., Beijing, China. The entire animal experiment complied with the requirements of the Animal Management and Ethics Committee of Jilin Agricultural University and followed the National Guiding Principles for the Welfare of Laboratory Animals strictly. If the animal developed dyspnea, hemorrhagic diarrhea, or showed signs of mortality, they were euthanized immediately by CO_2_ inhalation.

### Construction of recombinant *L. plantarum*

We constructed the recombinant *L. plantarum* expressing FomA protein by first synthesizing the FomA gene sequence (restriction endonuclease site: HindIII and XbaI; GenBank accession number: X72583). (A) We combined 5 μL of plasmid linked with the target fragment and 100 μL of NC8 competent cells in an electroporation cuvette, and placed it on ice for 5 min. (B) We selected the electroporation program 4-1-9, that is, the standard electroporation procedure for plant lactic acid bacteria; after the electroporation was completed, we let it stand at room temperature for 3 min. (C) We added 600 μL of MRS culture medium to the electroporation cuvette, mixed well, and then sucked the culture medium into an Eppendorf (EP) tube; we then incubated it on a 37°C shaker for 2 h. (D) We centrifuged the EP tube at 4,000 rpm for 2 min, removed the supernatant, leaving 100 μL to spread on an MRS solid culture plate with EM resistance, and incubated it overnight. The next day, we picked single colonies for identification. Gene Pulser Xcell Total System #1652660 (Bio-Rad Laboratories, Inc., USA). This FomA gene fragment was then inserted into the pSIP409-pgsA' and pSIP409-FnBPA-pgsA' plasmids and the resulting positive plasmid was transformed into the *L. plantarum* strain NC8 (CCUG 61730).

Two recombinant *L. plantarum* strains were obtained as follows: NC8-pSIP409-pgsA'-FomA and NC8-pSIP409-FnBPA-pgsA'-FomA. These were identified by sequencing carried out by Shanghai Shenggong Biotechnology Co., Ltd. (Shanghai, China).

### Preparation of the anti-FomA antibody

The FomA gene sequence was ligated into the pET28a expression vector and transformed into *Escherichia coli* BL21, generating recombinant *E. coli* BL21-pET-28a-FomA. FomA protein expression was induced with IPTG (100 mM; Sigma, Japan), purified, and recovered, and rabbits were immunized with purified FomA protein to obtain a rabbit polyclonal antibody for detecting the expression of bacterial target genes.

### Western blotting

To examine the expression of the FomA antigen, NC8-pSIP409-pgsA', NC8-pSIP409-FnBPA-pgsA', NC8-pSIP409-pgsA'-FomA, and NC8-pSIP409-FnBPA-pgsA'-FomA were collectively grown with 10 μg/ml erythromycin and 50 ng/ml Sakacin P inducer (SppIP) for 9 h at 30°C. Bacterial proteins separated by SDS-PAGE (10% acrylamide) were transferred to a nitrocellulose membrane and incubated with a polyclonal rabbit anti-FomA antibody and a goat anti-rabbit secondary antibody conjugated with horseradish peroxidase (HRP; Proteintech Group). The washed protein was visualized on the Amersham Imager (General Electric Company) via enhanced chemiluminescence (ECL, USA).

### Flow cytometry and immunofluorescence detection for recombinant *L. plantarum*

Plant bodies (0.5 ml) induced as described above were resuspended in PBS, diluted to the appropriate OD value, and incubated with the FomA antibody for 1 h at 4°C. Then, the samples were stained with FITC-labeled goat anti-mouse IgG (Proteintech Group) for 1.5 h at 4°C. Recombinant bacterial cells were evaluated by flow cytometry (BDLSR Fortessa cells, USA) and fluorescence microscopy (Leica DMI8, Germany).

### Animal-combined infection model of *F. nucleatum* and DSS

For *F. nucleatum*, 38.5 g of brain extract broth medium was weighed with a balance, dissolved in 1,000 ml distilled water, and transferred in anaerobic screw bottles. High-purity nitrogen was used to remove the oxygen from the test tube and medium, was autoclaved at 121°C for 15 min, restored to room temperature, and transferred to a 37° anaerobic workstation. Overall, frozen *F. nucleatum* solution (−80°C) was removed and 100 microliters were inoculated into 50 ml screw bottles under sterile conditions. After 8 h, the bacterial liquid was cloudy and removed from the anaerobic workstation, and the number of bacteria per fed was 5 × 10^8^.

For model construction, 32 mice were randomly divided into four groups, with eight mice in each group. The first group was administered PBS, the second group received an aqueous solution containing 2.5% DSS, the third group was administered *F. nucleatum*, and the fourth group received both *F. nucleatum* and an aqueous solution containing 2.5% DSS. Mice were euthanized after 8 days.

### Immunization and animal experiments

To assess the antiviral efficacy of *L. plantarum*, 32 C57BL/6 mice were randomly divided into four groups. The groups of mice were immunized with 200 μl of PBS, NC8, NC8-pSIP409-pgsA'-FomA, and NC8-pSIP409-FnBPA-pgsA'-FomA suspension containing colony-forming units (5 × 10^8^ CFU), and the control group received 200 μl of 0.9% normal saline in the same method. Oral immunization was performed via gavage. Mice were initially immunized on the 1st, 2nd, and 3rd days, followed by booster immunizations on the 12th, 13th, and 14th days, and further booster immunizations on the 24th, 25th, and 26th days. Feces and serum of mice were collected at days 0, 12, 24, and 36 and stored at −80°C.

After completion of immunization in the four groups, the protective effect of recombinant *L. plantarum* was assessed by administering DSS and *F. nucleatum*. Mice were observed daily, and changes in body weight and mortality were recorded. All remaining mice were euthanized on day 8. Blood was collected from experimental mice by the orbital venous plexus; serum was isolated from blood and stored at −80°C. Samples were washed twice with cold PBS containing 1% phenylsulfonyl fluoride (PMSF), and then, the samples were centrifuged to harvest the supernatant and stored at −80°C. Additionally, under sterile conditions, portions of the colon, spleen, and small intestine were randomly retained from the infected and control groups.

### Immune cell extraction from the intestine of a mouse

(A) Mice were euthanized, and the entire intestinal segment of the small intestine was removed and placed in pre-cooled PBS at 4°C. Fat and Peyer's Patch (PP) nodes were removed first. The intestinal tract was, then, dissected, washed, and cut into ~8-mm-sized fragments and collected in a centrifuge tube. A separation solution was added to separate the small intestinal epithelial cells and villi at 37°C and centrifuged at 180 rpm for 25 min. (B) The intestinal segments were collected again in a 50 ml centrifuge tube and placed in a 37°C incubator at 250 rpm for 25 min. The required amount of digestive solution was added based on the effect of digestion and the amount of tissue. During digestion, each chamber was shaken for 30 s within 10 min. After digestion, the solution was vigorously shaken for 1 min, then filtered into a 50 ml centrifuge tube using a 70 μm cell sieve, collected on ice, and supplemented with PBS to stop the reaction. The remaining intestinal tissue was put into a 50 ml centrifuge tube with 5 ml of digestive juice to continue digestion until thoroughly digested, typically requiring three treatments. (C) The single-cell suspension resulting from the multiple digestion steps was collected in 50 ml centrifuge tubes. Cells were re-precipitated using FACS buffer or PBS. The suspension was, then, carefully layered onto 80 and 40% percoll solution. Lymphocytes were, then, carefully aspirated, and the cells were resuspended in PBS solution. (D) The lymphocytes (white mist middle layer) were carefully aspirated with a pipette, and the aspirated cells were fed into PBS solution and centrifuged at 1,800 rpm, after removing the supernatant.

### Lymphocyte proliferation test

To evaluate the proliferation of primary immunized lymphocytes, MLN cells (2 × 10^5^ cells) and SLP cells (2 × 10^5^ cells) were placed in 96-well plates, and p54 antigen (5 μg/ml) was added to each well. After 72 h, the cells were removed, and 20 μl of MTS was added to each well to stop the reaction. After 4 h, the OD value was detected by an enzyme labeling instrument (Chen et al., 2021).

### Flow cytometry

Mice from each group were euthanized on day 12 after the last immunization (Huang et al., 2018). Single-cell suspensions from the spleens, mesenteric lymph nodes (MLNs), and Peyer's patches (PPs) were prepared from all groups of mice. Cells were stained using a mouse antibody (BD Biosciences, USA; Chen et al., 2021). BD fluorescence-activated cells were sorted and analyzed by FACS in an LSRFortessa analyzer (BD Bioscience, USA).

Single-cell suspensions from MLNs and PPs were prepared, and 10 μl of anti-B220 antibody (BD Bioscience, USA) was added to a test tube containing 1 × 10^6^ cells. The cells were mixed and stained in the dark at 4°C for 30 min, followed by the addition of 1 ml of phosphate-buffered saline (PBS). The cell suspension was centrifuged at 2,000 rpm and 4°C for 5 min, and the supernatant was discarded. The above steps were repeated. The cells were, then, fixed and permeabilized, centrifuged twice, and stained with 10 μl of anti-IgA antibody in the dark at 4°C for 30 min (BD Bioscience, USA). Single-cell suspensions of the MLNs and spleens were transferred to a 48-well cell culture plate, and the cells were incubated with the p54 protein for 8 h. Then, inhibitors were added for 3 h. The cells were centrifuged twice, and 10 μl of anti-CD3, anti-CD4, and anti-CD8 antibodies (BD Biosciences, USA) were added. The cells were, then, fixed, permeabilized, centrifuged twice, and mixed with 10 μl of anti-IFNγ, anti-Foxp3, anti-IL4, anti-IL10, anti-IL17, anti-IL22, and anti-IL13 antibodies (BD Biosciences, USA) for 30 min at 4°C in the dark. BD fluorescence-activated cells were sorted and analyzed by FACS in an LSRFortessa analyzer (BD Bioscience, USA). All data were analyzed using FlowJo10.8.1 software. The gating strategies for flow cytometry experiments are presented in Supplementary Figure 4.

### Enzyme-linked immunosorbent assay

Total IgA, IgG, IL6, IL1β, and TNFα levels in the gut and serum were determined using a commercially available ELISA kit (Jiangsu Meimian Industrial Co., Ltd., MEIMAN, China). Using the concentration of the standard substance as the x-axis and the OD value as the y-axis, a standard curve was drawn, then the OD value was entered for calculation, and the changes were observed in mouse.

### Pathological sections

Histopathological analysis was carried out on the small intestine, colon, and spleen samples collected after 8 days of infection. All samples were fixed with 4% paraformaldehyde, and sections were stained with hematoxylin and eosin to examine pathological changes.

### Statistical analyses

Data were analyzed with Prism 8 software (GraphPad, La Jolla, CA, United States) and were expressed as the mean ± standard deviation of at least three independent experiments. Differences between the groups were evaluated by one-way analysis of variance. A *P*-value of < 0.05 was considered statistically significant.

## Results

### Construction of plasmids and *in vitro* expression of target genes

Initially, we obtained a 1,140 bp FomA fragment (Supplementary Figure 1A), which was then ligated to pET-28a and transferred to BL21 for prokaryotic expression (Supplementary Figure 1B). This process validated that a rabbit-specific antibody was procured by immunizing rabbits with the purified FomA antigen. Subsequently, we inserted the resulting FomA fragments into two plasmids, pSIP409-pgsA' and pSIP409-FnBPA-pgsA', respectively (Supplementary Figures 1C, D). The FomA gene was accurately identified via restriction enzyme digestion and confirmed by nucleic acid electrophoresis and sequencing results. As a result, we successfully acquired two recombinant plasmids, pSIP409-pgsA'-FomA and pSIP409-FnBPA-pgsA'-FomA (Supplementary Figures 1E, F). FomA gene order, Comments for pSIP409 and primer-related files (Supplementary material).

**Figure 1:**
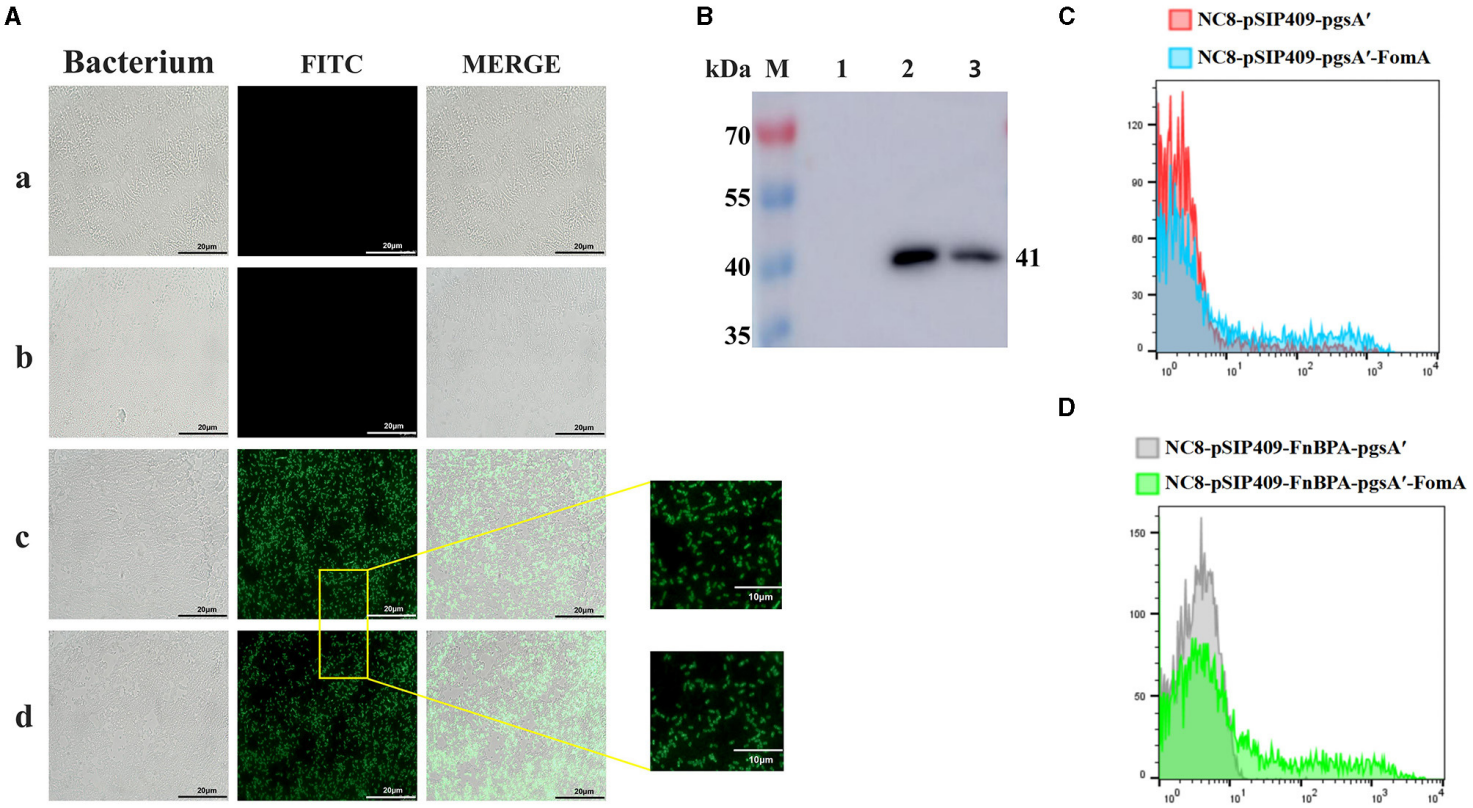
Expression of the FOMA genes from recombinant *L. plantarum* NC8-pSIP409-pgsA'-FomA and NC8-pSIP409-FnBPA-pgsA'-FomA. **(A)** Immunofluorescence experiments to verify the expression of the FomA protein on the surface of the *L. plantarum*. a: NC8-pSIP409-pgsA'. b: NC8-pSIP409-FnBPA-pgsA'. c: NC8-pSIP409-pgsA'-FomA. d: NC8-pSIP409-FnBPA-pgsA'-FomA. **(B)** The expression of the FomA protein in the recombinant *L. plantarum* NC8-pSIP409-pgsA'-FomA and NC8-pSIP409-FnBPA-pgsA'-FomA was verified by Western blot. M: protein Marker; lane 1: NC8; lane 2: NC8-pSIP409-pgsA'-FomA; lane 3: NC8-pSIP409-FnBPA-pgsA'-FomA. **(C)** FomA protein expression in the recombinant *L. plantarum* NC8-pSIP409-pgsA'-FomA was verified by flow cytometry. **(D)** FomA protein expression in the recombinant *L. plantarum* NC8-pSIP409-FnBPA-pgsA'-FomA was verified by flow cytometry.

The recombinant plasmid was introduced into NC8 via electroconversion. To verify the surface expression of the target protein, we performed immunofluorescence experiments (Figure 1A). We detected the expression of FomA via Western blot experiments (Figure 1B), which revealed that the expression of NC8-pSIP409-pgsA'-FomA and NC8-pSIP409-FnBPA-pgsA'-FomA at 41 kDa matched the size of FomA, while NC8-pSIP409-pgsA' and NC8-pSIP409-FnBPA-pgsA' showed no FomA protein expression. Flow cytometry, consistent with immunofluorescence and Western blot results, indicated no significant expression in the control group, while a distinct positive peak was evident in the recombinant *L. plantarum* (Figures 1C, D). These results confirm the successful construction and expression of the FomA protein in two recombinant *L. plantarum* NC8-pSIP409-pgsA'-FomA and NC8-pSIP409-FnBPA-pgsA'-FomA.

### Immunization procedures and co-infection experiments

To assess the enhanced immune efficacy of recombinant *L. plantarum* in mice and prevent co-infection with *F. nucleatum* and dextran sulfate sodium (DSS), we designed an animal experiment (Supplementary Figure 2). Animals were divided into four groups as follows: two control groups (PBS and NC8) and two experimental groups (recombinant *L. plantarum* NC8-pSIP409-pgsA'-FomA and NC8-pSIP409-FnBPA-pgsA'-FomA). After oral immunization, some mice were designated for immune effect evaluation, while others were assigned for protection experiments to assess resistance to severe IBD caused by *F. nucleatum* infection.

### Recombinant *L. plantarum* induces activation of immune cells

**Figure 2:**
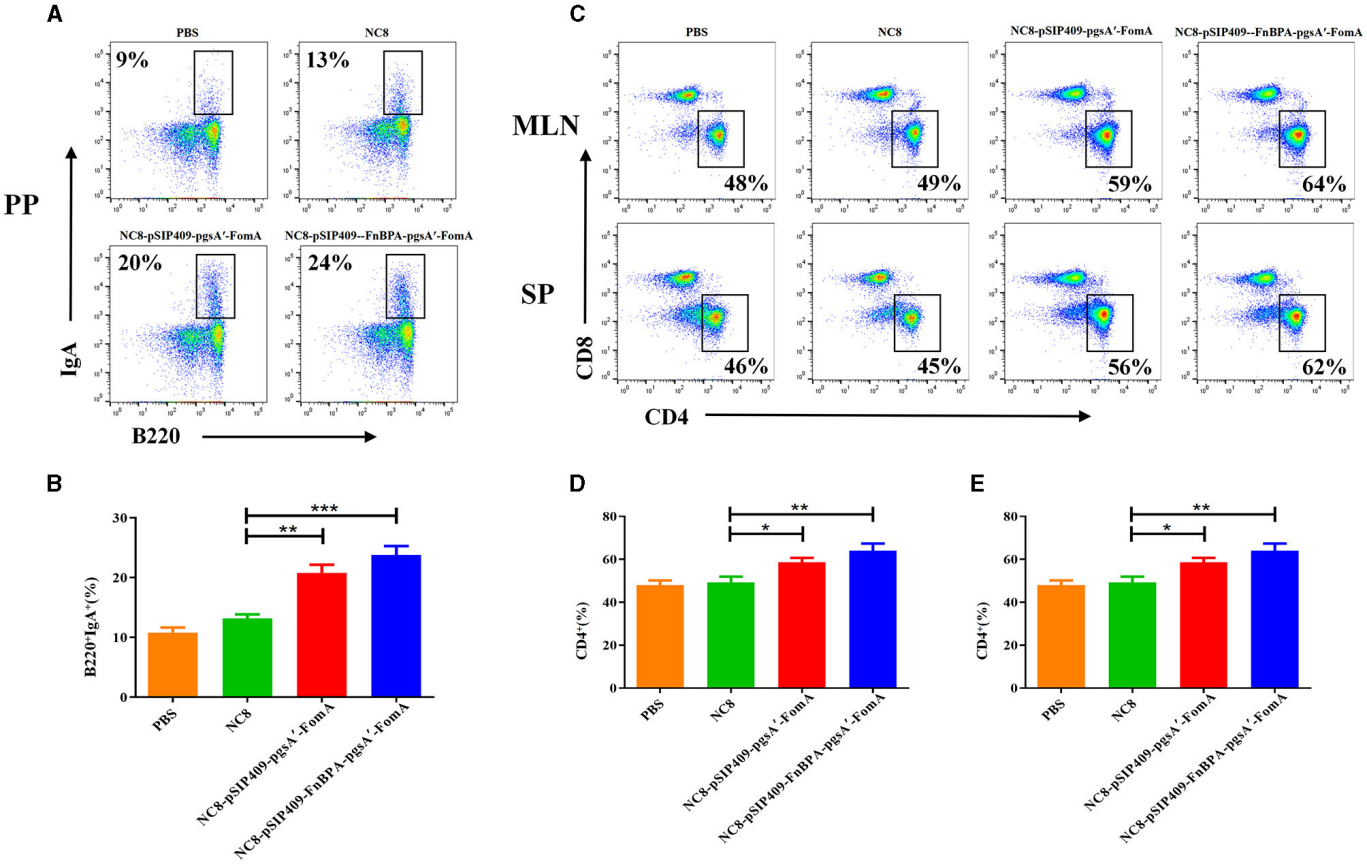
Flow cytometry analysis of B and T cells in mouse PPs, MLN, and SP. **(A)** Flow cytometry results of lymphocyte B220 and IgA expression in mouse PPs. **(B)** Statistical analysis of lymphocyte expression of B220 and IgA in mouse PPs. **(C)** Changes in the detection of CD3^+^ CD4^+^ and CD3^+^ CD8^+^ T cells in the SP and MLN. **(D)** Statistical analysis of lymphocyte expression of CD3^+^ CD4^+^ T cells in the MLN. **(E)** Statistical analysis of lymphocyte expression of CD3^+^ CD4^+^ T cells in the SP. ^*^p < 0.05, ^**^p < 0.01, ^***^p < 0.001.

Intestinal B cells, capable of regulating the intestinal microecological balance by secreting IgA, play a pivotal role in the specific binding to intestinal bacteria (Lycke and Bemark, 2017). We observed that recombinant *L. plantarum* significantly augmented the detection of B220^+^IgA^+^ cells in Peyer's Patches (PPs). Compared with the control group, the experimental group demonstrated a significant increase in the number of B cells in the PPs and a substantial elevation in IgA secretion (Figure 2A). Notably, the difference was even more pronounced in mice fed with NC8-pSIP409-FnBPA-pgsA'-FomA as compared with those fed with NC8 (Figure 2B).

**Figure 3:**
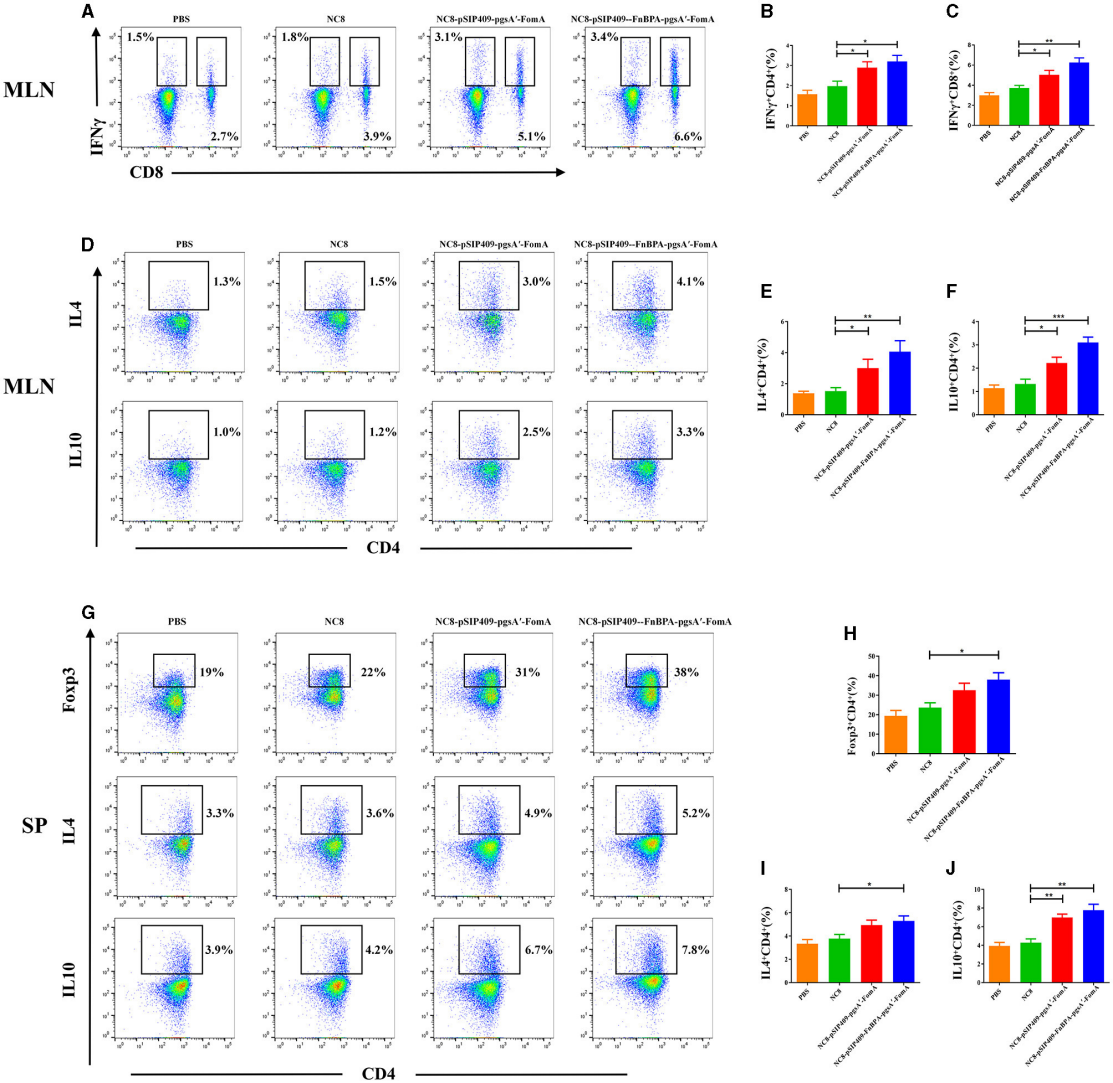
Cytokine expression in T cells was further analyzed by flow cytometry. **(A)** Flow cytometry results of lymphocyte IFNγ expression in mouse MLN. **(B)** Statistical analysis of lymphocyte expression of CD4 and IFNγ in mouse MLN. **(C)** Statistical analysis of lymphocyte expression of CD8 and IFNγ in mouse MLN. **(D)** Results of IL4 and IL10 expression in CD4 T cells in mouse MLN by flow cytometry. **(E)** Statistical analysis of CD4 T cells expression of IL4 in mouse MLN. **(F)** Statistical analysis of CD4 T cells expression of IL10 in mouse MLN. **(G)** Results of Foxp3, IL4, and IL10 expression in CD4 T cells in mouse SP by flow cytometry. **(H)** Statistical analysis of CD4 T cell expression of Foxp3 in mouse SP. **(I)** Statistical analysis of CD4 T cell expression of IL4 in mouse SP. **(J)** Statistical analysis of CD4 T cell expression of IL10 in mouse SP. ^*^p < 0.05, ^**^p < 0.01, ^***^p < 0.001.

For the T-cell analysis, the activation was considerable in the experimental group. In both the mesenteric lymph nodes (MLNs) and spleen (SP), the proportion of CD4^+^T cells significantly escalated (Figure 2C). Particularly, the activation of CD4^+^T cells was more pronounced in the mice fed with NC8-pSIP409-FnBPA-pgsA'-FomA as compared with those fed with NC8 (Figures 2D, E). We also found a notable increase in the secretion of IFNγ by CD8^+^T cells in the MLN of mice from the NC8-pSIP409-FnBPA-pgsA'-FomA group and a similar elevation in IFNr secretion by CD8-T cells (CD3^+^CD8^−^T cells, mainly Th1; Figures 3A–C).

### Activation of CD4^+^T cells induced by recombinant *L. plantarum*

Significantly activated CD4^+^T cells were further examined. In the MLN, mice immunized with recombinant *L. plantarum* NC8-pSIP409-FnBPA-pgsA'-FomA and NC8-pSIP409-pgsA'-FomA strongly stimulated the secretion of IL4 and IL10 in comparison to the control group (Figure 3D). The levels of IL4 and IL10 in the invasive NC8-pSIP409-FnBPA-pgsA'-FomA group were higher than the other three groups (Figures 3E, F).

In the SP, we noted a significant increase in the content of Treg cells in the experimental group compared with the control group (Figures 3G, H). Moreover, the secretion of IL4 and IL10 by CD4^+^ T cells also significantly escalated (Figures 3G, I, J). These results indicate that the NC8-pSIP409-FnBPA-pgsA'-FomA induced a significant Th1/2 mixed immune response in MLN and SP.

### Immunoglobulin secretion elevated by recombinant *L. plantarum*

**Figure 4:**
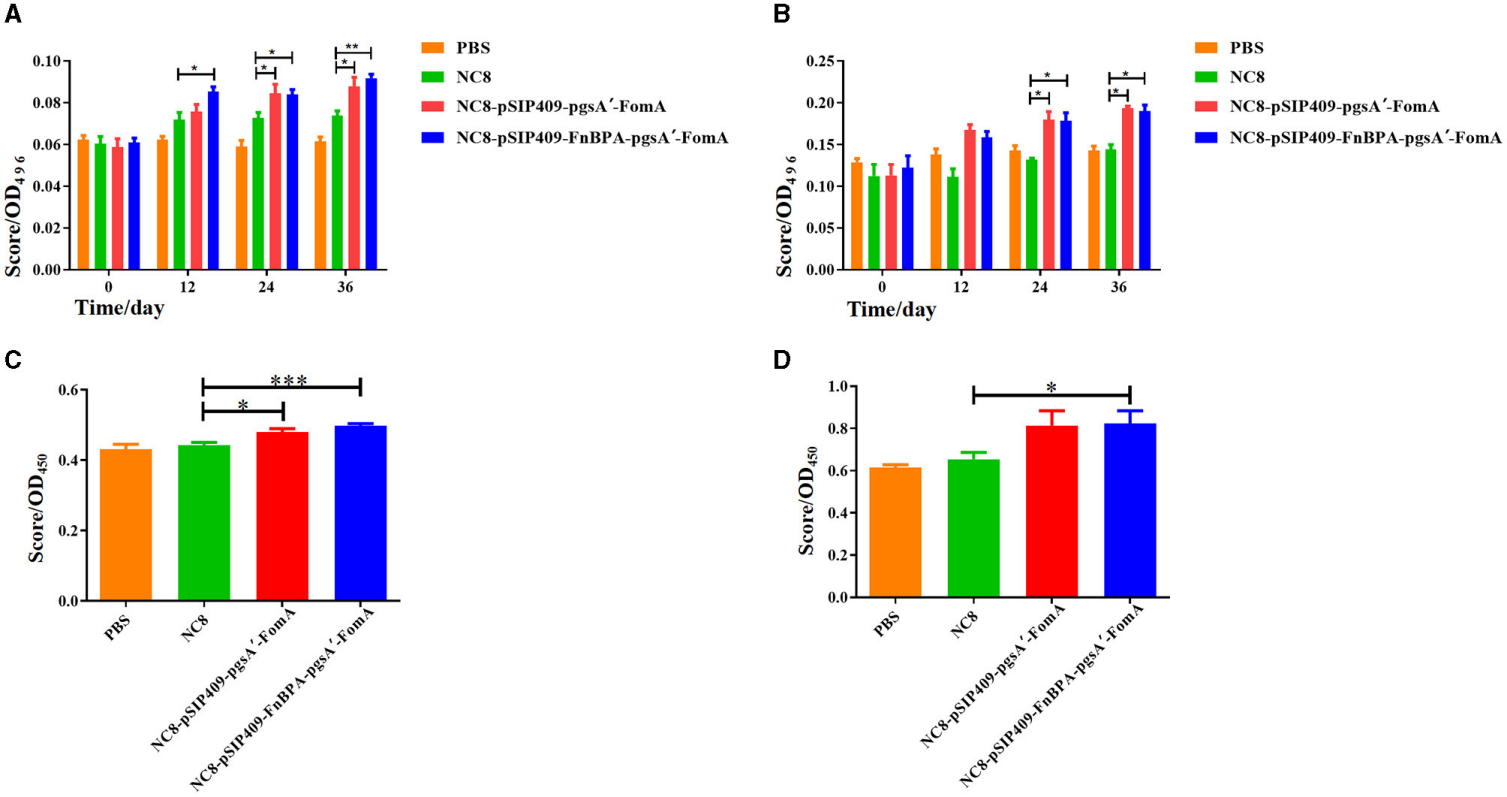
Results of the ELISA experiments **(A)** Comparison of the increase in the fecal IgA content of the four groups of mice after each immunization. **(B)** The increase in the blood serum IgG content of the four groups of mice after each immunization. **(C)** The results of lymphocyte proliferation in the MLN. **(D)** The results of lymphocyte proliferation in the SP. ^*^p < 0.05, ^**^p < 0.01, ^***^p < 0.001.

The secretory IgA (SIgA) tests in mouse feces showed that prior to immunization, SIgA content was similar across all groups (Figure 4A). After the first immunization, mice fed with NC8-pSIP409-FnBPA-pgsA'-FomA exhibited significantly higher SIgA content in feces than control mice. This trend was more evident after the second and third immunizations, with the highest SIgA amount observed in mice fed with NC8-pSIP409-FnBPA-pgsA'-FomA. While SIgA levels in PBS mice did not show any significant change after three immunizations, the SIgA content was found to be increased in the feces of NC8, NC8-pSIP409-pgsA'-FomA, and NC8-pSIP409-FnBPA-pgsA'-FomA mice, with the most substantial difference in the NC8-pSIP409-FnBPA-pgsA'-FomA group.

Similarly, serum IgG content was similar in all groups before immunization (Figure 4B). However, the post-immunization period witnessed a significant surge in IgG content in the experimental mice, especially after the third immunization. The concentration of IgG antibodies in PBS mouse serum was 79.2 ng/mL; in the NC8 group, it was 81.8 ng/ml; in the NC8-pSIP409-pgsA'-FomA group mouse serum, it was 110.5 ng/ml; and in the NC8-pSIP409-FnBPA-pgsA'-FomA group mouse serum, it was 114.3 ng/ml. The mice fed with NC8-pSIP409-pgsA'-FomA and NC8-pSIP409-FnBPA-pgsA'-FomA had considerably higher IgG levels than that of other groups. These results demonstrate an increase in systemic immunity by recombinant *L. plantarum*.

Importantly, our results show an increased SIgA secretion in the feces, which signifies that the recombinant *L. plantarum* effectively induces local mucosal immunity.

### Cellular immune response induced by recombinant *L. plantarum*

The results of the MLN lymphocyte proliferation showed that mice fed with recombinant *L. plantarum* NC8-pSIP409-pgsA'-FomA and NC8-pSIP409-FnBPA-pgsA'-FomA demonstrated a significant proliferation compared with those fed with PBS and NC8 (Figure 4C). A similar trend was observed in the SP lymphocyte proliferation, with the group fed with NC8-pSIP409-FnBPA-pgsA'-FomA showing greater proliferation than others (Figure 4D). These results indicate the existence of memory lymphocytes in the experimental mice group, which proliferate and produce specific antibodies upon antigen stimulation. It was also found that memory lymphocytes in MLN play a more substantial role in this immune response.

### Recombinant *L. plantarum* alleviates severe IBD induced by *F. nucleatum*

Initially, a mouse model of severe IBD caused by *F. nucleatum* was established. Feeding DSS and *F. nucleatum* to mice resulted in severe IBD symptoms. By day 5, mice in the DSS and *F. nucleatum* groups exhibited slight anal bleeding, while the other groups did not show significant changes. By day 7, severe anal injuries and bleeding were observed in the DSS and *F. nucleatum* group. Concurrently, a decline in the survival rate, severe weight loss, and significant colon atrophy was observed in this group (Supplementary Figure 3).

**Figure 5:**
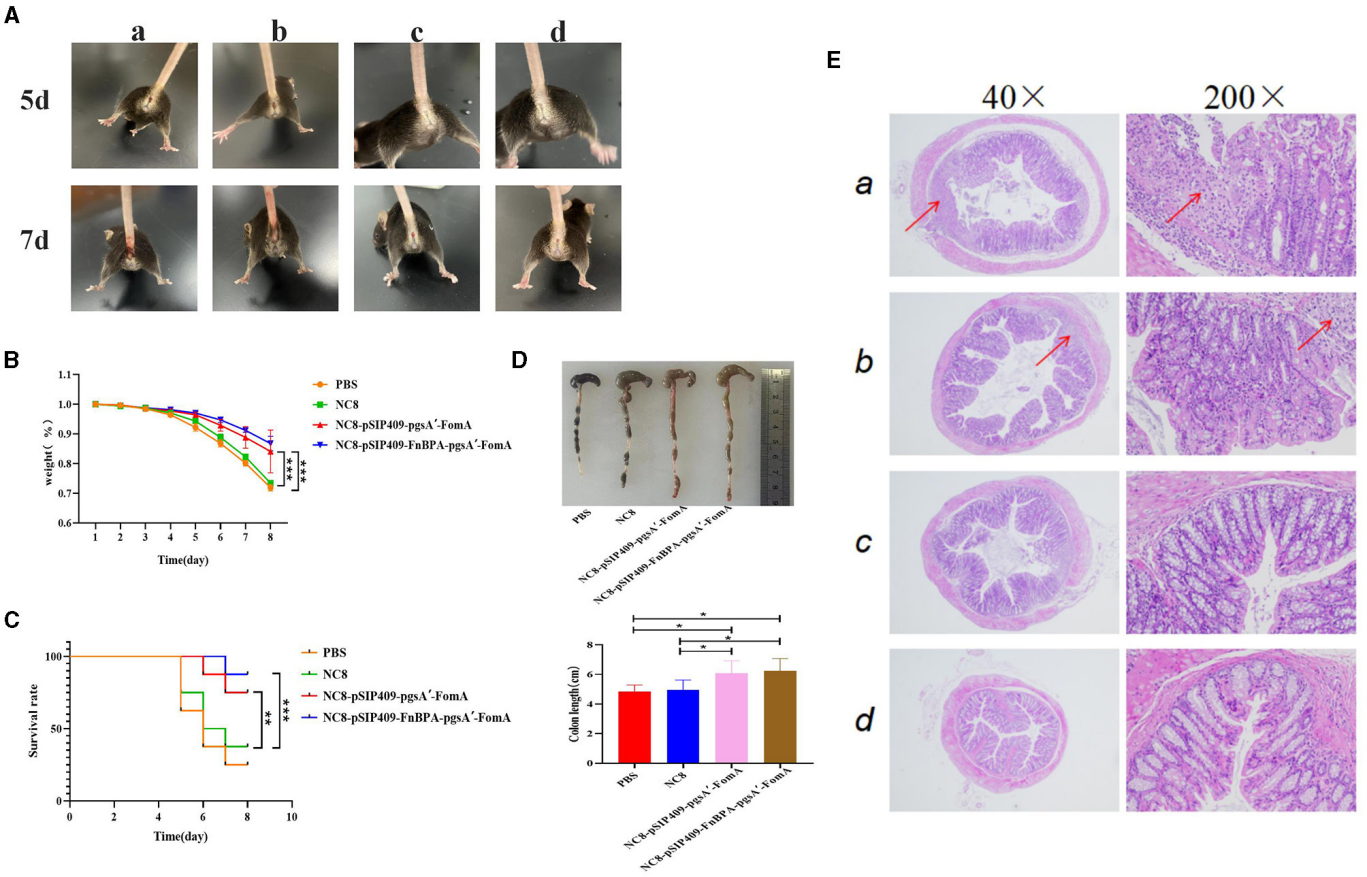
Feeding recombinant *L. plantarum* can prevent the combined infection of *F. nucleatum* and DSS. **(A)** Combined infection of *F. nucleatum* and DSS. *F. nucleatum* co-infection with DSS on days 5 and 7. a: PBS b: NC8 c: NC8-pSIP409-pgsA'-FomA. d: NC8-pSIP409-FnBPA-pgsA'-FomA. **(B)** Statistics of body weight loss rate in mice. **(C)** Statistics of the mouse survival curves. **(D)** Statistics of the colon length in mice. **(E)** Pathological sections of the mouse colon are displayed. a: PBS b: NC8 c: NC8-pSIP409-pgsA'-FomA. d: NC8-pSIP409-FnBPA-pgsA'-FomA. ^*^p < 0.05, ^**^p < 0.01, ^***^p < 0.001.

For the four groups of mice that had undergone three oral immunizations, a co-infection of *F. nucleatum* and DSS was performed. On the 5th day, significant anal bleeding was observed in the mice fed with both PBS and NC8 (Figure 5A). By day 7, severe symptoms were present in the anus of the PBS and NC8 groups, while the NC8-pSIP409-pgsA'-FomA and NC8-pSIP409-FnBPA-pgsA'-FomA groups showed no particularly significant anal pathology. By day 8, the mortality rate and body weight changes were analyzed. Mice in the NC8-pSIP409-pgsA'-FomA and NC8-pSIP409-FnBPA-pgsA'-FomA groups had the lowest mortality rate and less body weight loss compared with the PBS and NC8 groups, which displayed high mortality rates and drastic body weight changes (Figures 5B, C). Histological examination revealed severe colon atrophy in the control group (Figure 5D). Under 40x magnification, severe pathological changes were observed in the colons of mice fed with PBS and NC8, with structural and morphological changes in the intestines and diffused intestinal cell walls. In contrast, the colon of mice fed with recombinant *L. plantarum* showed no significant inflammation. Under 200x magnification, severe damage and shedding of the intestinal villus were observed in the PBS and NC8 groups, while the NC8-pSIP409-pgsA'-FomA group displayed mild pathological changes with only partial loss of intestinal villus epithelial cells. Notably, no significant histopathological changes were observed in the colon of mice in the NC8-pSIP409-FnBPA-pgsA'-FomA group (Figure 5E).

In conclusion, the study demonstrates that recombinants *L. plantarum* NC8-pSIP409-pgsA'-FomA and NC8-pSIP409-FnBPA-pgsA'-FomA can significantly alleviate severe IBD symptoms caused by *F. nucleatum*.

### Immune cells and cytokine detection in the intestine and serum of mice

**Figure 6:**
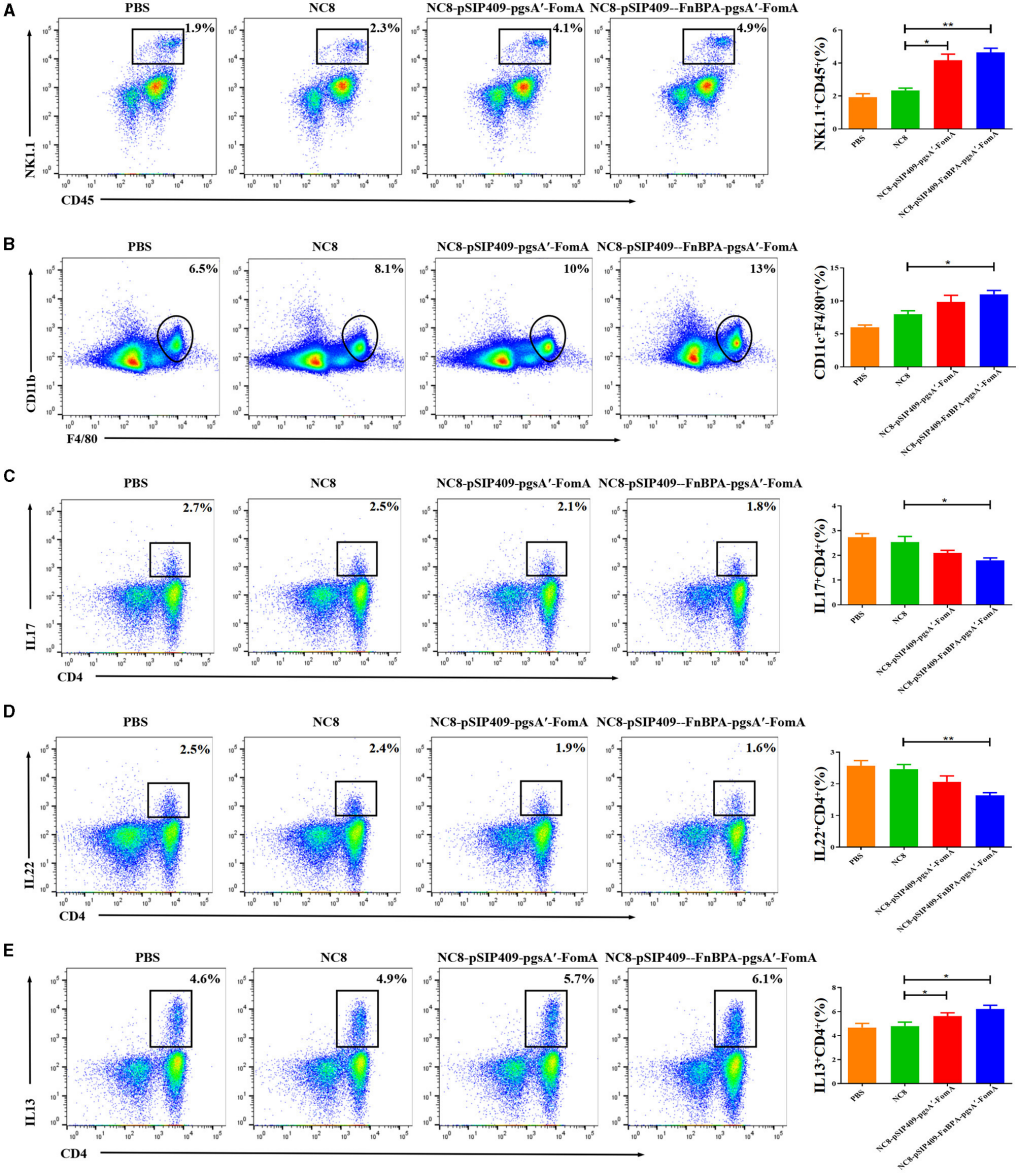
**(A)** Statistics of the differential NK cells in the blood of the four mouse groups. **(B)** Statistics of differences in macrophages in blood from the four mouse groups. **(C)** Differential statistics of IL 17 secreted by CD4^+^ T cells in the jejunum of the four mouse groups. **(D)** Differential statistics of IL 22 secreted by CD4^+^ T cells in the jejunum of the four mouse groups. **(E)** Differential statistics of IL 13 secreted by CD4^+^ T cells in the jejunum of the four mouse groups. ^*^p < 0.05, ^**^p < 0.01.

The flow cytometry results showed significant activation of NK cells (Figure 6A) and macrophages (Figure 6B) in mice fed with recombinant *L. plantarum* groups compared with the PBS and NC8 groups. Moreover, the secretion of IL17 (Figure 6C) and IL22 (Figure 6D) by CD4^+^T cells was notably reduced in these mice, while the secretion of IL13 was significantly increased (Figure 6E). Serum analysis revealed higher secretions of IgG and IgA (Supplementary Figures 4A, B) and the cytokines IL6 and IL1β (Supplementary Figures 4C, D) in the experimental group compared with the control group. Conversely, the secretion of TNFα was lower in the experimental group (Supplementary Figure 4E). The cytokine levels in the colon of the experimental group were similar to those in the serum. However, the secretion of IL6 and IL1β was more significantly elevated in the colon of the experimental group compared with the control group (Supplementary Figures 4F, G), and the secretion of TNFα was significantly reduced (Supplementary Figure 4H).

In summary, the data suggest that feeding mice with recombinant *L. plantarum* leads to significant changes in immune cell activation and cytokine secretion, both in the intestine and serum. The experimental group displayed enhanced activation of NK cells and macrophages, along with an increase in IL13, IgG, and IgA secretion. At the same time, they showed a decrease in IL17, IL22, and TNFα secretion. This indicates that recombinant *L. plantarum* potentially modulates the immune response and contributes to the regulation of inflammatory responses in severe IBD caused by *F. nucleatum*.

## Discussion

*F. nucleatum*, a symbiotic bacterium in humans and animals, primarily colonizes the oral cavity and colon mucosa. Co-infection with inflammatory bowel disease (IBD) in the colon can be severely harmful to patients (Castellarin et al., 2012; Brennan and Garrett, 2019; Engevik et al., 2021). This highlights the need for the rational development of a protective vaccine. FomA, an important outer membrane protein in *F. nucleatum*, has immunogenic properties and can activate the immune response of the body, thereby influencing host invasion (Nakagaki et al., 2010; Martin-Gallausiaux et al., 2020; Zhang et al., 2022). The recombinant *L. plantarum* surface expression vector used in this experiment is mature and has been utilized in developing various new vaccines, achieving satisfactory preventive effects (Chen et al., 2021; Xue et al., 2022). In this study, the FomA antigen elicited a strong immune response in mice, providing preventive effects against the co-infection of *F. nucleatum* and DSS.

When *Lactococcus lactis* expresses FnBPA on its surface, it can bind to host cell surface receptors, thereby allowing recombinant bacteria to infect cells and achieve the delivery of exogenous DNA (Innocentin et al., 2009). In a mouse model, the recombinant *Lactococcus lactis* can express FnBPA, increasing plasmid transfer in the mouse intestine, proving that these recombinant *Lactococcus lactis* strains can be well-applied to DNA transfer (Almeida et al., 2014). pValac-GFP is a eukaryotic shuttle plasmid synthesized in the laboratory. The recombinant invasive *Lactococcus lactis* can deliver exogenous DNA through this plasmid and express this DNA in epithelial cells (Guimarães et al., 2009). In one study, the pValacDNA eukaryotic expression vector encoding IL10 was electroporated into *Lactococcus lactis* expressing FnBPA, and it was found that the recombinant strain could effectively inhibit the inflammatory response in a mouse colitis model (Zurita-Turk et al., 2014). Liu et al. used *L. plantarum* to express FnBPA, creating a new type of invasive lactic acid bacteria, which showed better adhesion and invasion abilities in pig intestinal epithelial cells (IPEC-J2) and could stimulate BMDC differentiation and IL6 and MyD88 mRNA expression. As such, when FnBPA is located on the surface of *L. plantarum*, it substantially enhances the invasion efficiency of somatic cells. FNBPA has been used as an adjuvant to co-express exogenous antigens to boost the immune response of the body and has achieved significant effects.

Mucosal immunization offers potential advantages over conventional parenteral immunization by eliciting immune defense in both mucosal and systemic tissues to protect against pathogen invasion at mucosal surfaces (Li et al., 2020). Vaccines, using recombinant *L. plantarum* administered mucosally, can promote the production of mucosal IgA or SIgA, effectively activating the mucosal immune response (Boyaka, 2017; Jin et al., 2019). Mucosal IgA or SIgA is structurally resistant to chemical degradation in the harsh environment of mucosal surfaces and enzymes of host or microbial origin. For example, the human mucosal anti-influenza vaccine induced higher local humoral and cellular immune responses (Calzas and Chevalier, 2019). In our study, we found that feeding mice with recombinant *L. plantarum* not only promoted the content of SIgA produced in the intestinal mucosa but also enhanced IgA B-cell surface responses in Peyer's patches (PPs).

IgG is a crucial antibody in the body performing functions such as antiviral, neutralizing viruses, antibacterial, and immune regulation (Barabas et al., 2018). Our study showed that IgG levels in the blood increased significantly after each immunization, especially after 12 days of second and third immunizations. Mice fed with NC8-pSIP409-pgsA'-FomA and NC8-pSIP409-FnBPA-pgsA'-FomA reached high levels of IgG (Sitt et al., 2019). Therefore, the increased IgG content in body fluids can enhance the activity against *F. nucleatum*.

During the immunization process with recombinant *L. plantarum*, immune cells, including CD4 T cells in both the mesenteric lymph nodes (MLNs) and spleen (SP), showed significant proliferative activation. These activated CD4 T cells include various subsets, such as Treg, Th1, and Th2. Our further analysis revealed a significant increase in the secretion of Th1 cytokine IFNγ and Th2 cytokines IL4 and IL10 in the MLN of the experimental group compared with the control group. Similarly, in the SP of the experimental group of mice, we observed a significant increase in the proportion of Treg cells, along with a significant increase in cytokines secreted by Th1/2 cells. These results suggest that feeding mice with recombinant *L. plantarum* can enhance the activation of CD4 T cells and the secretion of Th1/2 cytokines in MLN and SP. Furthermore, *in vitro* cell proliferation experiments indicated the generation of memory lymphocytes in both MLN and SP after three oral immunizations, which could be activated upon antigenic stimulation.

We used a DSS solution and *F. nucleatum* to construct an experimental model in mice (Lin et al., 2022). Studies suggest that the co-infection of DSS and *F. nucleatum* can exacerbate IBD, leading to intestinal structure destruction and abnormal cytokine secretion (Yu et al., 2020; Yamashita et al., 2021). Our experimental results indicate that feeding mice with recombinant *L. plantarum* could mitigate the severity of IBD caused by co-infection, providing some level of protection in terms of survival rate, weight loss rate, and pathological changes.

Natural killer (NK) cells demonstrate natural cytotoxicity and are vital immune cells capable of eliminating tumor cells and virus-infected cells by directly destroying them. NK cells play a crucial role in regulating various hematopoietic, inflammatory, and immune responses (O'brien and Finlay, 2019). Macrophages play a vital role in the innate response to pathogens and the initiation of inflammation (Artyomov et al., 2016). Regulatory T (Treg) cells modulate immune responses during intestinal inflammation, and pro-inflammatory factors IL17 and IL22 are majorly secreted by CD4^+^T cells (Schmitt et al., 2021). IL13, primarily derived from CD4^+^T lymphocytes, controls inflammatory responses by inhibiting LPS-induced mononucleotide release (Iwaszko et al., 2021). Our results indicate that feeding mice with recombinant *L. plantarum* resulted in the activation of NK cells and macrophages in the intestine, while CD4^+^T cells demonstrated decreased expression of IL17 and IL22 and increased expression of IL13.

IgA and IgG are the most abundant immunoglobulins in the human body playing a critical role in mucosal immunity, development of tolerance, and protection against infections (Singh et al., 2014). Following an inflammatory response, a diverse and complex network of cytokines is produced. Among them, TNFα is central to the inflammatory response, with high blood TNFα levels indicative of an inflamed state in the body (Cruceriu et al., 2020). IL1β, an important agonist of the IL1 family, is a multifunctional cytokine, which is crucial for activating neutrophils and macrophages to clear invading pathogens, thereby serving as a vital component of host defense against infection (Lopez-Castejon and Brough, 2011). Rapid production of IL6 contributes to host defense during infection and tissue damage. We discovered that feeding mice with recombinant *L. plantarum* not only significantly increased the secretion of cytokines such as IgA, IgG, IL6, and IL1β in the serum and IL6 and IL1β in the colon but also intriguingly led to a significant decrease in TNFα secretion both in serum and the colon.

In this study, we created recombinant *L. plantarum* NC8-pSIP409-pgsA'-FomA and NC8-pSIP409-FnBPA-pgsA'-FomA expressing the important outer membrane protein FomA of *F. nucleatum*. As a result of the persistent surface response and the presence of the exogenous protein FomA in the intestine of mice, a protective immune response was elicited in the mice. This response included, but was not limited to, the promotion of humoral and cellular immunity. Both recombinant strains protected mice from severe IBD caused by *F. nucleatum*. Though we evaluated numerous protective indicators that substantiated improvements in mouse immunity, our study has its limitations.

Immune evaluation results from the mouse model showed that recombinant *L. plantarum* NC8-pSIP409-FnBPA-pgsA'-FomA could more effectively activate humoral, cellular, and mucosal immunity in mice. Our findings hold significant implications for the development of a novel oral immune vaccine aimed at mitigating severe IBD due to *F. nucleatum* infection.

## Conclusion

In conclusion, our results demonstrate that oral administration of recombinant *L. plantarum* NC8-pSIP409-FnBPA-pgsA'-FomA significantly stimulates the mucosal and cellular immune responses in mice, promotes immunoactivation of induced Th1/type 2 cells, and enhances the secretion of cytokines such as IgA, IgG, IL6, and IL1β. This, in turn, alleviates the combined infection of *F. nucleatum* and DSS. These findings provide evidence for a novel type of oral recombinant *L. plantarum* vaccine.

## Data availability statement

The original contributions presented in the study are included in the article/Supplementary material, further inquiries can be directed to the corresponding author.

## Ethics statement

The animal study was approved by the Animal Management and Ethics Committee of Jilin Agricultural University. The study was conducted in accordance with the local legislation and institutional requirements.

## Author contributions

XZ and YJ conceived and designed the research. XZ and HX conducted experiments. HZ analyzed data. XZ wrote the manuscript. All authors contributed to the article and approved the submitted version.
