# Supplementary material for: Lactobacillus plantarum surface-displayed FomA (Fusobacterium nucleatum) protein generally stimulates protective immune responses in mice
Source: Front Microbiol. 2023 Sep 20;14:1228857. doi: 10.3389/fmicb.2023.1228857 (PMC10548212; doi:10.3389/fmicb.2023.1228857)
Supplement: Supplementary file 1 [file Data_Sheet_1.pdf]

1. FomA gene order

The gene sequence of the FomA protein (NCBI number: X72583.2) was searched through the NCBI website for the following base codes.

ATGAAAAAATTAGCATTAGTATTAGGTTTATTATTAGTAGTTGGATCAGTTGC
ATCAGCTAAAGAAGTTATGCCTGCACCTACTCCAGCTCCTGAAAAAGTAGT
AGAATATGTTGAAAAACCAGTTATAGTTTACAGAGACAGAGAAGTTGCTCC
AGCTTGGAGACCAAATGGTTCAGTAGATGTTCAATACAGATGGTATGGAGA
AGTTGAAAAGAAAAATCCAAAAGATGATAAAGATGAAAACTGGGCAACTG
GTAAAGTAAATGCCGGAAGATTACAAACTTTAACAAAAGTAAACTTTACTG
AAAAACAAACTTTAGAAGTAAGAACAAGAAATCATCATACTTTAAATGATA
CAGATGCAAATAACAAGAAATCAAATGGAGCAGCTGATGAATATAGATTAA
GACACTTCTATAACTTTGGAAAGTTAGGTTCATCTAAAGTTAATGCTACTTC
AAGAGTAGAATTTAAACAAAAAACAAATGATGGAGAAAAATCTTTAGGAG
CATCAGTATTATTTGATTTTGCTGATTATATCTATTCTAACAATTTCTTTAAAG
TTGATAAATTAGGATTAAGACCAGGATATAAATATGTATGGAAAGGACATGG
AAATGGTGAAGAAGGAACTCCTACAGTTCATAATGAATATCATTTAGCATTT
GAATCTGATTTCACATTACCATTTAACTTTGCTTTAAACTTAGAATATGATTT
ATCTTATAACAGATATAGAGAAAAATTCGAAACAACAGATGGATTAAAGAA
AGCTGAATGGTATGGTGAATTAACAGCTGTACTTTCTAACTACACTCCATTAT
ATAAAGCAGGGGCATTTGAATTAGGATTCAATGCTGAAGGTGGATATGATAC
TTACAATATGCACCAATATAAGAGAATTGGTGGAGAAGATGGAACTTCTGTT
GACAGAAGAGACTATGAATTATACTTAGAACCAACTCTACAAGTTTCTTATA
AACCAACAGATTTCGTAAAACTATATGCAGCAGCAGGAGCTGACTACAGAA
ATAGAATTACTGGAGAATCAGAAGTTAAGAGATGGAGATGGCAACCAACTG
CTTCGGCTGGTATGAAAGTTACTTTCTAA

2. FomA gene sequence optimization

To better express FomA protein, we optimized the gene sequence of TomA and added the restriction site sequence and HA tag gene in Shanghai Biotech Company, and the synthetic sequence results are as follows.

TCTAGAATGTACCCATACGATGTTCCAGATTACGCTAAAAAACTGGCGCTGG
TTCTGGGTCTGCTGCTGGTTGTTGGTTCTGTTGCGAGCGCGAAAGAAGTTA
TGCCGGCGCCGACCCCGGCGCCGGAAAAAGTTGTTGAATATGTTGAAAAA
CCGGTTATCGTTTACCGTGATCGTGAAGTTGCGCCGGCGTGGCGTCCGAAC
GGTAGCGTTGATGTTCAGTACCGTTGGTACGGTGAAGTTGAAAAGAAAAA
CCCGAAAGATGATAAAGATGAAAACTGGGCTACCGGTAAAGTGAACGCGG
GCCGTCTGCAGACCCTGACCAAAGTTAACTTCACCGAAAAACAGACCCTG
GAAGTTCGTACCCGTAACCACCACACCCTGAACGATACCGATGCTAACAAC
AAAAAATCTAACGGCGCGGCTGATGAATACCGTCTGCGTCACTTCTACAAC
TTCGGTAAACTGGGCAGCTCTAAAGTTAACGCTACCTCTCGTGTTGAATTC
AAACAGAAAACCAACGATGGTGAAAAAAGCCTGGGCGCGAGCGTTCTGTT

CGATTTCGCGGATTACATCTACTCTAACAACTTCTTCAAAGTTGATAAACTG
GGTCTGCGTCCGGGCTACAAATACGTGTGGAAAGGCCACGGTAACGGTGA
AGAAGGTACCCCGACTGTTCACAACGAATACCACCTGGCGTTCGAATCTGA
TTTCACCCTGCCGTTCAACTTCGCGCTGAACCTGGAATACGATCTGAGCTA
CAACCGTTACCGTGAAAAATTCGAAACCACCGATGGCCTGAAAAAAGCGG
AATGGTACGGTGAACTGACCGCGGTTCTGAGCAACTACACCCCGCTGTATA
AAGCGGGCGCATTCGAACTGGGTTTCAACGCGGAAGGCGGCTACGACACC
TACAACATGCACCAGTACAAACGTATCGGTGGTGAAGATGGTACCAGCGTT
GATCGTCGTGATTACGAACTGTACCTGGAACCGACCCTGCAGGTTTCCTAC
AAACCGACCGATTTCGTTAAACTGTACGCGGCAGCGGGTGCGGATTACCGT
AACCGTATCACCGGTGAATCTGAAGTTAAACGTTGGCGTTGGCAGCCGACC
GCGAGCGCGGGTATGAAAGTTACCTTCTAAAAGCTT

3. Comments for pSIP409

Puc is an essential gene for replication in E. coli, 256 rep is a necessary gene for replication in Lactobacillus NC8, And ermL is the erythromycin resistance gene, PsppIP Is the gene inducing the expression of SPPPK and SPPR genes, SPPK and SPPR can further induce the expression of the target genes, Tcat194 and TsaiA are terminator, Porfx is the promoter to induce the expression of the target genes, The TpepN is the terminator.

4. Primer design

To construct the E. coli expression vector for FomA gene fragments with restriction sites XhoI and NdeI, upstream and downstream primers were first designed for PCR amplification according to the FomA gene sequence, retaining the promoter, removing the terminator, adding the NdeI restriction site at the upstream 5' end and the XhoI restriction site at the downstream 5' end.

F:CGCATAATGTACCCATACGATGTTCCAGATT

R:CTCGAGTTAGAAGGTAACTTTCATACCCGC
