# Supplementary material for: Lactobacillus plantarum surface-displayed FomA (Fusobacterium nucleatum) protein generally stimulates protective immune responses in mice
Source: Front Microbiol. 2023 Sep 20;14:1228857. doi: 10.3389/fmicb.2023.1228857 (PMC10548212; doi:10.3389/fmicb.2023.1228857)

Figure S1 |Construction of recombinant plasmids pSIP409-pgsA'-FomA and pSIP409-FnBPA-pgsA'-FomA. (A) The synthesis of the FomA gene (1140bp) was verified by nucleic acid electrophoresis. (B) The expression of the FomA protein was examined by Western blot (C) Identification of the FomA insert in plasmids extracted from randomly picked pSIP409-pgsA′-FomA colonies. lane 1: plasmids digested with KpnI/XbaI; lane 2: pSIP409-pgsA′-FomA plasmid. (D) Identification of the FomA insert in plasmids extracted from randomly picked pSIP409-FnBPA-pgsA'-FomA colonies. lane 1: plasmids digested with HindIII/XbaI; lane 2: pSIP409-FnBPA-pgsA′′-FomA plasmid. (E) A map of the plasmid pSIP409-pgsA'-FomA (F) a map of the plasmid pSIP409-FnBPA-pgsA'-FomA.

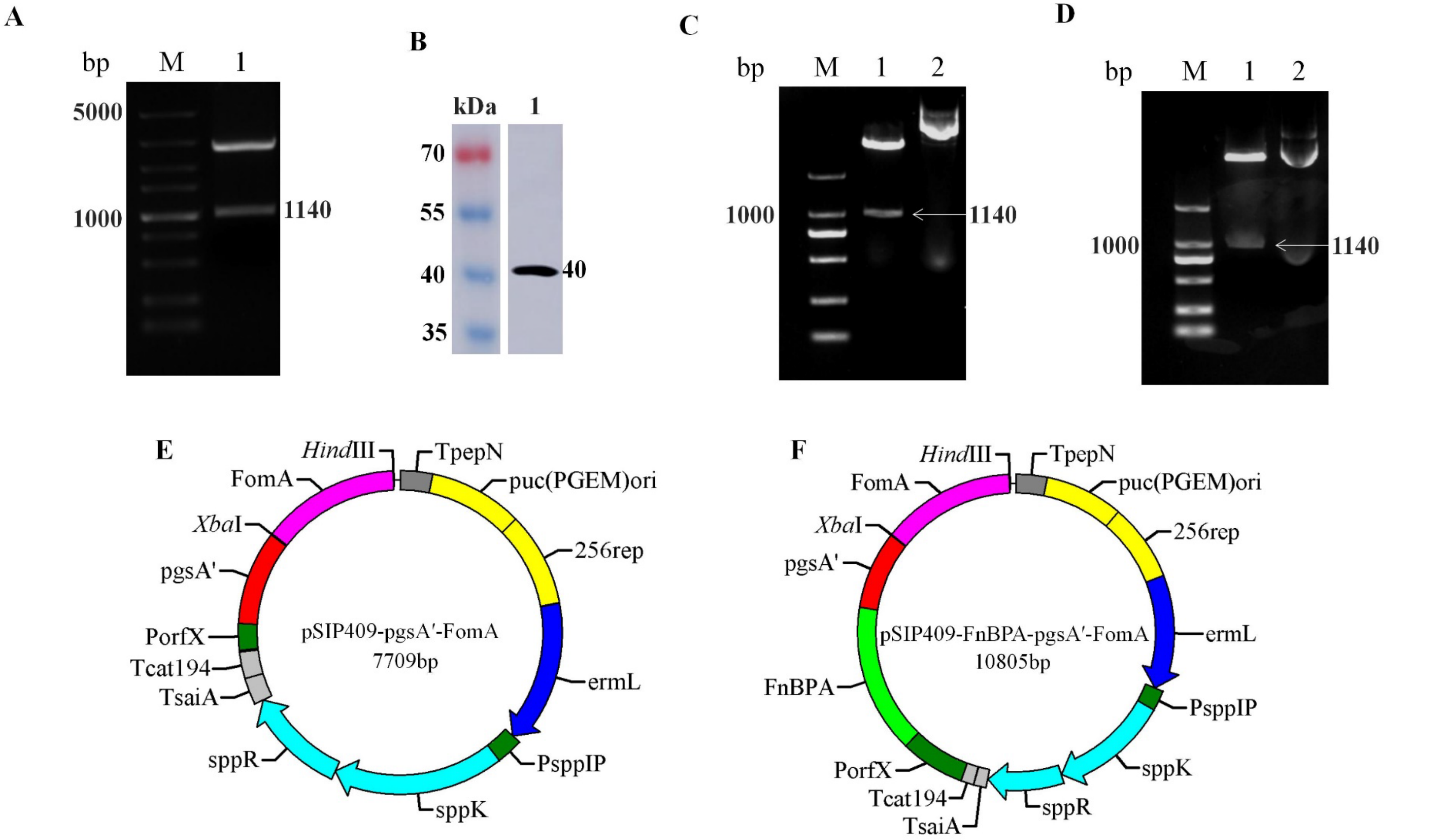

Figure S2 | Experimental design of mice. Mice were immunized with recombinant L. plantarum on days 1–3,12–14 and 24–26. On day 36 using a combination of F.nucleatum and DSS. Mouse immune cells were harvested on day 36 for in vitro experiments. ELISA was performed on days 0,12,24,36, and 44. Changes in mice were recorded after the combination of F.nucleatum and DSS treatment and pathological examination was performed on day 8. Final evaluation of the recombinant L. plantarum vaccine against coinfection with F.nucleatum and DSS.

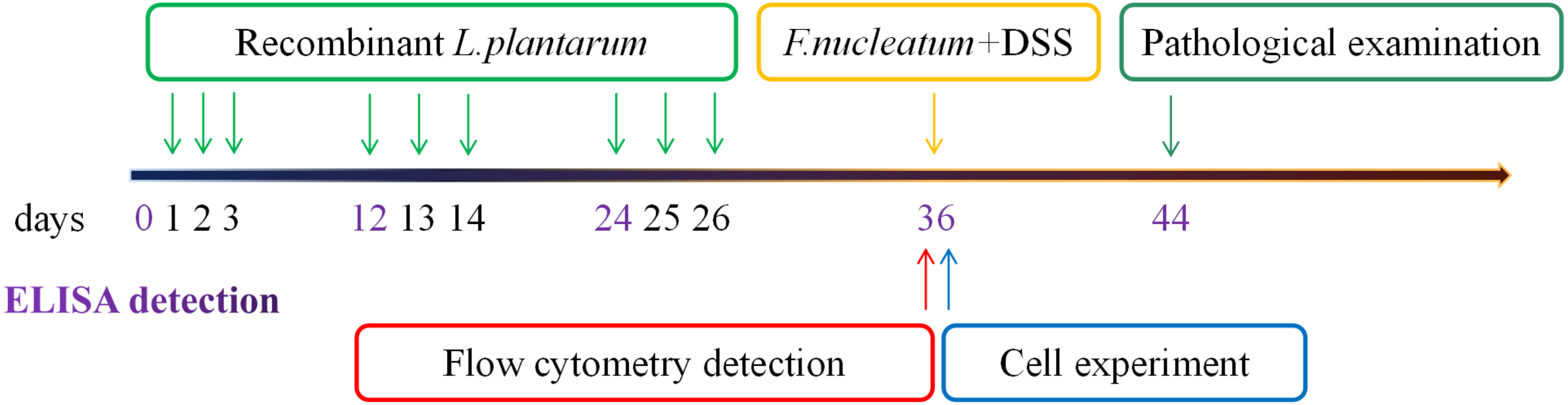

Figure S3 | Results of mouse model of inflammatory bowel disease (A) Production of anal blood stools in mice fed *F. nucleatum* and aqueous solution containing 2.5% DSS (left for day 5, right for day 7, a: DSS+*F. nucleatum* b: DSS) (B) Mouse survival curves for the four groups (C) Body weight change curves of the four groups (D) Colon length statistics for the four mouse groups

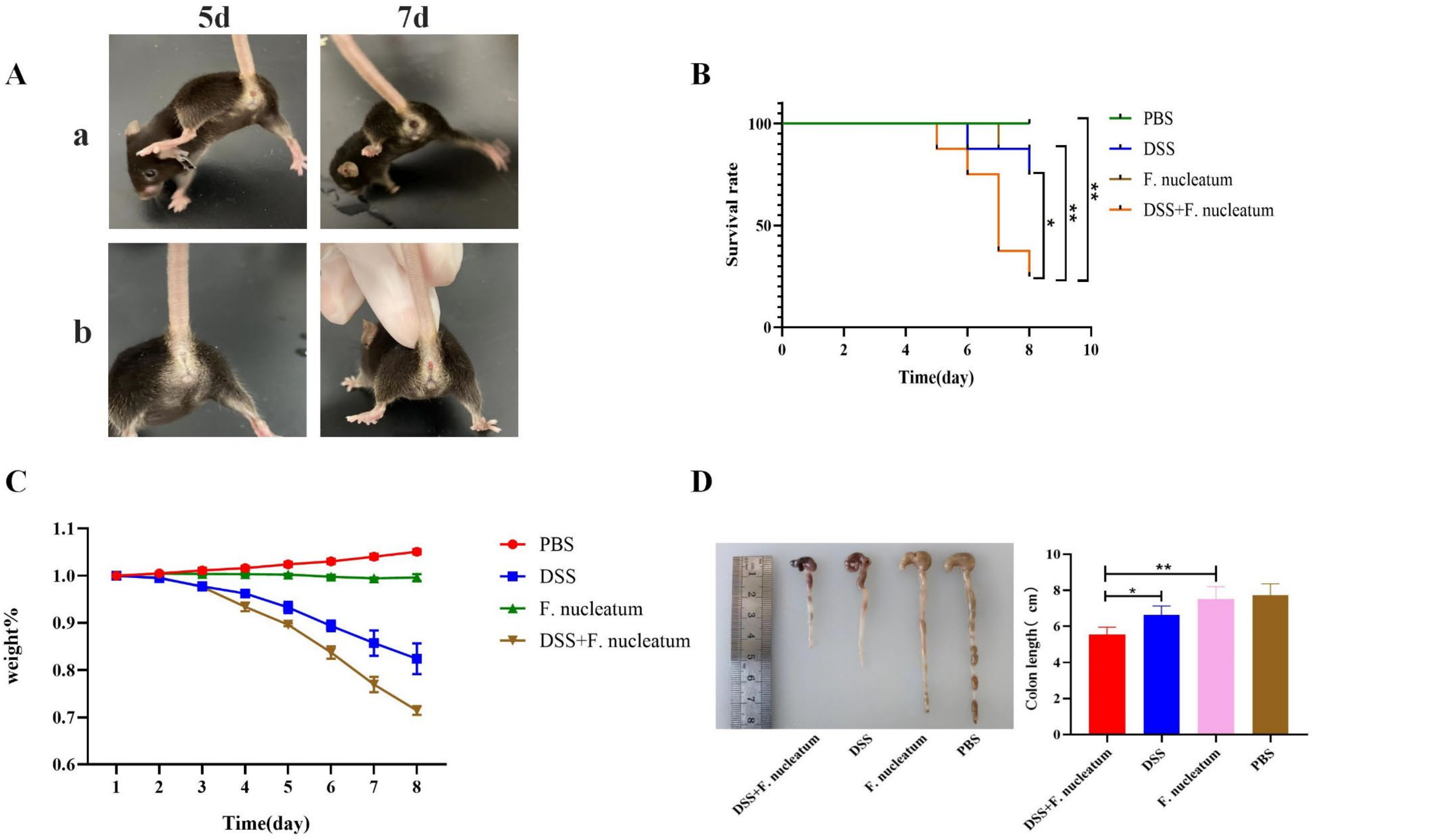

Figure S4 | Statistics of cytokine secretion in the colon and serum after day 8 of mouse infection. (A) Of the IgA in the serum. (B) Of the IgG in the serum. (C) Of the IgG in the serum. (D) Of the IL6 in the serum. (E) Of the IL1β in the serum. (F) Of the TNFα in the colon. (G) Of the IgG in the colon. (H) Of the IgG in the colon.

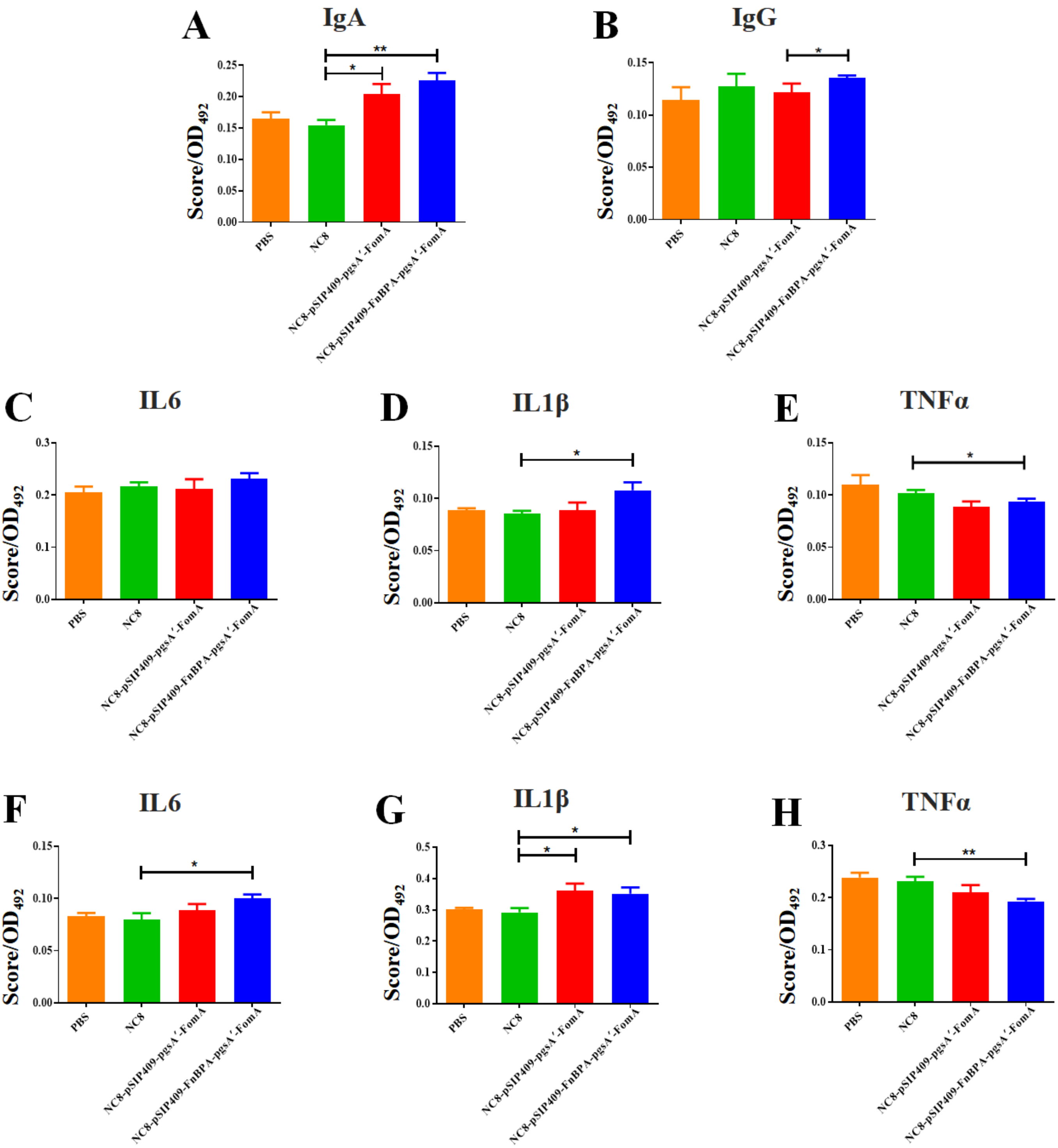

Figure S5 | The gating strategies for flow cytometry experiments. (A) The gating strategies of MLN; SP and PP. Lymphocytes were first selected and then removed by FSCA and FSCH before selection for B cell analysis / T cell analysis. (B) The gating strategies of LPL, Lymphocytes were first selected and then removed by FSCA and FSCH before selection for Macrophages/T cell analysis.

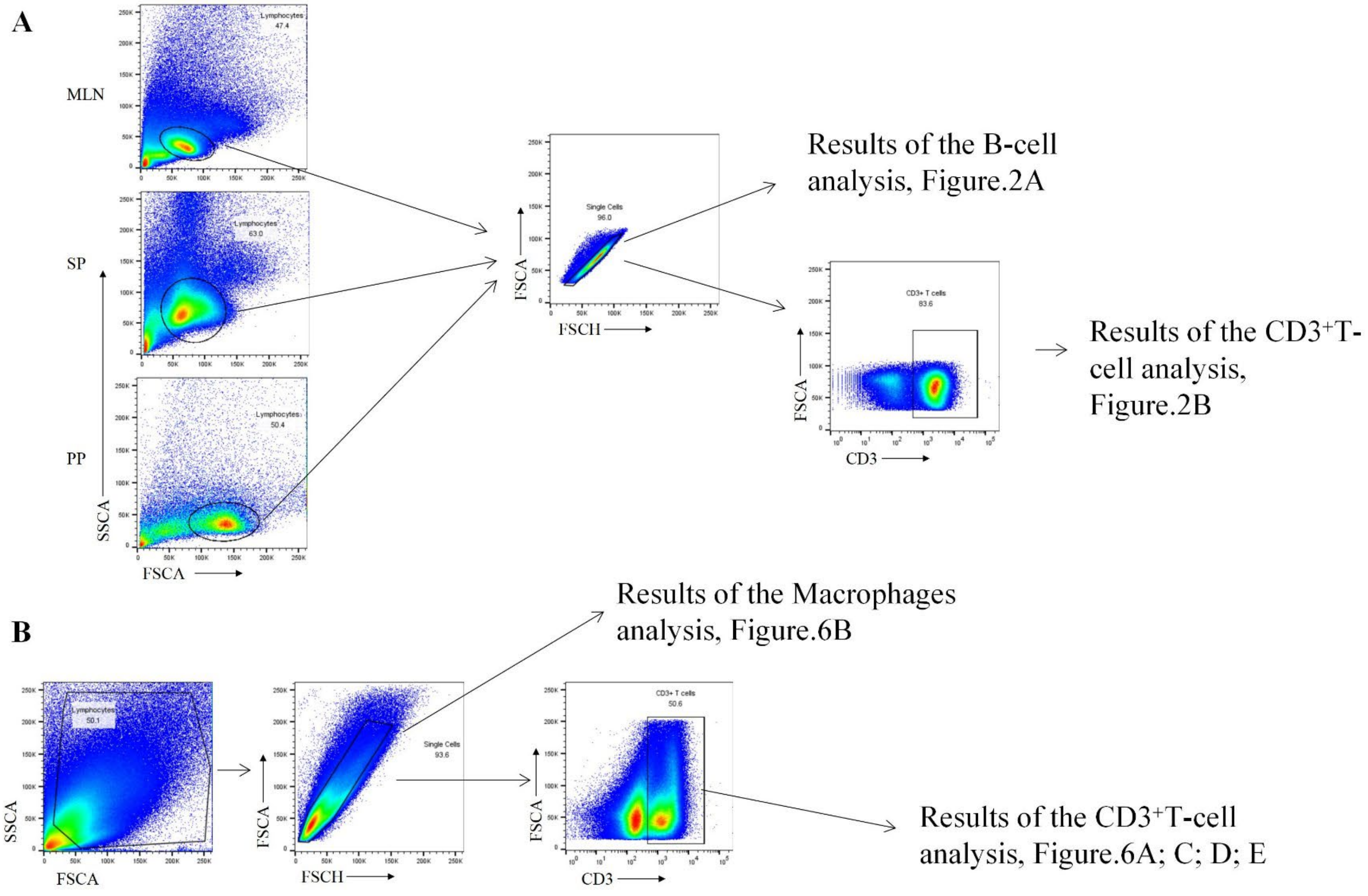

Supplement: Supplementary file 2 [file Data_Sheet_2.pdf]
